# Supplementary material for: Discovery and application of insertion-deletion (INDEL) polymorphisms for QTL mapping of early life-history traits in Atlantic salmon
Source: BMC Genomics. 2010 Mar 8;11:156. doi: 10.1186/1471-2164-11-156 (PMC2838853; doi:10.1186/1471-2164-11-156)
Supplement: Additional file 2 — Information on developed 76 locus single-run INDEL panel in Atlantic salmon. Information on fluorescence labeling, primer concentrations, PCR pooling and links to alignments, INDEL motifs and GENESCAN (Burge and Karlin 1997) predictions of genes/exons are available in html format. [file 1471-2164-11-156-S2.ZIP › Additionalfile2/table15402.html]

Contig Alignment

```
Base	A B C D E F   
1	G . . . . .    
2	G . . . . .    
3	G . . . . .    
4	G . . . . .    
5	A . . . . .    
6	G . . . . .    
7	A . . . . .    
8	G . . . . .    
9	T . . . . .    
10	G . . . . .    
11	G . . . . .    
12	G . . . . .    
13	A . . . . .    
14	C . . . . .    
15	A . . . . .    
16	G . . . . .    
17	C . . . . .    
18	T . . . . .    
19	G . . . . .    
20	C . . . . .    
21	T . . . . .    
22	A . . . . .    
23	T . . . . .    
24	G . . . . .    
25	T . . . . .    
26	A . . . . .    
27	C . . . . .    
28	C . . . . .    
29	A . . . . .    
30	T . . . . .    
31	C . . . . .    
32	A . . . . .    
33	A . . . . .    
34	G . . . . .    
35	G . . . . .    
36	A . . . . .    
37	C . . . . .    
38	A . . . . .    
39	C . . . . .    
40	G . . . . .    
41	C . . . . .    
42	A . . . . .    
43	G . . . . .    
44	C . . . . .    
45	A . . . . .    
46	G . . . . .    
47	T . . . . .    
48	T . . . . .    
49	C . . . . .    
50	C . . . . .    
51	T . . . . .    
52	G . . . . .    
53	C . . . . .    
54	A . . . . .    
55	G G G . . .    
56	T T T . . .    
57	C C C . . .    
58	C C C . . .    
59	T T T . . .    
60	T T T . . .    
61	C C C . . .    
62	C C C . . .    
63	A A A . . .    
64	G G G . . .    
65	C C C . . .    
66	T T T . . .    
67	C C C . . .    
68	C C C . . .    
69	A A A . . .    
70	G G G . . .    
71	A A A . . .    
72	G G G . . .    
73	C C C . . .    
74	C C C . . .    
75	C C C . . .    
76	T T T . . .    
77	G G G . . .    
78	G G G . . .    
79	G G G . . .    
80	A A A . . .    
81	G G G . . .    
82	A A A . . .    
83	G G G . . .    
84	A A A . . .    
85	C C C . . .    
86	A A A . . .    
87	G G G . . .    
88	C C C . . .    
89	A A A . . .    
90	G G G . . .    
91	G G G . . .    
92	C C C . . .    
93	A A A . . .    
94	A A A . . .    
95	G G G . . .    
96	A A A . . .    
97	C C C . . .    
98	C C C . . .    
99	T T T . . .    
100	G G G . . .    
101	G G G . . .    
102	T T T . . .    
103	G G G . . .    
104	C C C . . .    
105	T T T . . .    
106	G G G . . .    
107	C C C . . .    
108	A A A . . .    
109	G G G . . .    
110	G G G . . .    
111	A A A . . .    
112	G G G . . .    
113	A A A . . .    
114	G G G . . .    
115	A A A . . .    
116	G G G . . .    
117	T T T . . .    
118	C C C . . .    
119	A A A . . .    
120	C C C . . .    
121	A A A . . .    
122	C C C . . .    
123	T T T . . .    
124	G G G . . .    
125	C C C . . .    
126	A A A . . .    
127	G G G . . .    
128	C C C . . .    
129	T T T . . .    
130	C C C . . .    
131	C C C . . .    
132	G G G . . .    
133	C C C . . .    
134	T T T . . .    
135	C C C . . .    
136	A A A . . .    
137	G G G . . .    
138	C C C . . .    
139	A A A . . .    
140	C C C . . .    
141	G G G . . .    
142	T T T . . .    
143	T T T . . .    
144	A A A . . .    
145	T T T . . .    
146	G G G . . .    
147	A A A . . .    
148	G G G . . .    
149	G G G . . .    
150	T T T . . .    
151	C C C . . .    
152	C C C . . .    
153	A A A . . .    
154	G G G . . .    
155	G G G . . .    
156	A A A . . .    
157	C C C . . .    
158	A A A . . .    
159	T T T . . .    
160	C C C . . .    
161	T T T . . .    
162	T T T . . .    
163	C C C . . .    
164	T T T . . .    
165	G G G . . .    
166	T T T . . .    
167	C C C . . .    
168	T T T . . .    
169	G G G . . .    
170	T T T . . .    
171	C C C . . .    
172	A A A . . .    
173	G G G . . .    
174	C C C . . .    
175	A A A . . .    
176	G G G . . .    
177	G G G . . .    
178	A A A . . .    
179	G G G . . .    
180	A A A . . .    
181	C C C . . .    
182	A A A . . .    
183	G G G . . .    
184	G G G . . .    
185	A A A . . .    
186	T T T . . .    
187	G G G . . .    
188	C C C . . .    
189	A A A . . .    
190	G G G . . .    
191	A A A . . .    
192	T T T . . .    
193	C C C . . .    
194	A A A . . .    
195	T T T . . .    
196	C C C . . .    
197	A A A . . .    
198	G G G . . .    
199	C C C . . .    
200	T T T . . .    
201	G G G . . .    
202	G G G . . .    
203	G G G . . .    
204	A A A . . .    
205	C C C . . .    
206	A A A . . .    
207	G G G . . .    
208	A A A . . .    
209	C C C . . .    
210	A A A . . .    
211	G G G . . .    
212	C C C . . .    
213	T T T . . .    
214	G G G . . .    
215	G G G . . .    
216	C C C . . .    
217	C C C . . .    
218	A A A . . .    
219	G G G . . .    
220	G G G . . .    
221	G G G . . .    
222	A A A . . .    
223	C C C . . .    
224	A A A . . .    
225	G G G . . .    
226	G G G . . .    
227	G G G . . .    
228	A A A . . .    
229	G G G . . .    
230	A A A . . .    
231	T T T . . .    
232	G G G . . .    
233	A A A . . .    
234	A A A . . .    
235	A A A . . .    
236	C C C . . .    
237	G G G . . .    
238	C C C . . .    
239	A A A . . .    
240	A A A . . .    
241	G G G . . .    
242	A A A . . .    
243	C C C . . .    
244	T T T . . .    
245	G G G . . .    
246	G G G . . .    
247	G G G . . .    
248	G G G . . .    
249	A A A . . .    
250	A A A . . .    
251	A A A . . .    
252	C C C . . .    
253	G G G . . .    
254	G G G . . .    
255	G G G . . .    
256	G G G . . .    
257	T T T . . .    
258	C C C . . .    
259	G G G . . .    
260	A A A . . .    
261	C C C . . .    
262	C C C . . .    
263	A A A . . .    
264	G G G . . .    
265	G G G . . .    
266	G G G . . .    
267	G G G . . .    
268	G G G . . .    
269	A A A . . .    
270	A A A . . .    
271	G G G . . .    
272	A A A . . .    
273	G G G . . .    
274	T T T . . .    
275	T T T . . .    
276	C C C . . .    
277	T T T . . .    
278	C C C . . .    
279	T T T . . .    
280	G G G . . .    
281	T T T . . .    
282	C C C . . .    
283	A A A . . .    
284	G G G . . .    
285	C C C . . .    
286	T T T . . .    
287	G G G . . .    
288	C C C . . .    
289	T T T . . .    
290	A A A . . .    
291	C C C . . .    
292	A A A . . .    
293	C C C . . .    
294	A A A . . .    
295	G G G . . .    
296	A A A . . .    
297	A A A . . .    
298	A A A . . .    
299	G G G . . .    
300	C C C . . .    
301	C C C . . .    
302	T T T . . .    
303	T T T . . .    
304	G G G . . .    
305	G G G . . .    
306	A A A . . .    
307	G G G . . .    
308	A A A . . .    
309	G G G . . .    
310	G G G . . .    
311	A A A . . .    
312	A A A . . .    
313	G G G . . .    
314	A A A . . .    
315	G G G . . .    
316	A A A . . .    
317	G G G . . .    
318	G G G . . .    
319	A A A . . .    
320	G G G . . .    
321	T T T . . .    
322	C C C . . .    
323	A A A . . .    
324	T T T . . .    
325	G G G . . .    
326	A A A . . .    
327	T T T . . .    
328	G G G . . .    
329	A A A . . .    
330	G G G . . .    
331	G G G . . .    
332	A A A . . .    
333	A A A . . .    
334	A A A . . .    
335	G G G . . .    
336	C C C . . .    
337	G G G . . .    
338	G G G . . .    
339	C C C . . .    
340	A A A . . .    
341	G G G . . .    
342	A A A . . .    
343	G G G . . .    
344	G G G . . .    
345	G G G . . .    
346	G G G . . .    
347	C C C . . .    
348	A A A . . .    
349	G G G . . .    
350	A A A . . .    
351	G G G . . .    
352	G G G . . .    
353	G G G . . .    
354	G G G . . .    
355	G G G . . .    
356	A A A . . .    
357	C C C . . .    
358	T T T . . .    
359	G G G . . .    
360	G G G . . .    
361	A A A . . .    
362	G G G . . .    
363	C C C . . .    
364	T T T . . .    
365	G G G . . .    
366	T T T . . .    
367	G G G . . .    
368	G G G . . .    
369	T T T . . .    
370	T T T . . .    
371	G G G . . .    
372	C C C . . .    
373	T T T . . .    
374	G G G . . .    
375	G G G . . .    
376	G G G . . .    
377	G G G . . .    
378	C C C . . .    
379	A A A . . .    
380	G G G . . .    
381	A A A . . .    
382	G G G . . .    
383	G G G . . .    
384	G G G . . .    
385	G G G . . .    
386	A A A . . .    
387	A A A . . .    
388	C C C . . .    
389	G G G . . .    
390	T T T . . .    
391	C C C . . .    
392	G G G . . .    
393	T T T . . .    
394	G G G . . .    
395	A A A . . .    
396	A A A . . .    
397	G G G . . .    
398	C C C . . .    
399	A A A . . .    
400	G G G . . .    
401	A A A . . .    
402	C C C . . .    
403	A A A . . .    
404	C C C . . .    
405	G G G . . .    
406	T T T . . .    
407	G G G . . .    
408	G G G . . .    
409	G G G . . .    
410	T T T . . .    
411	C C C . . .    
412	T T T . . .    
413	T T T . . .    
414	T T T . . .    
415	T T T . . .    
416	C C C . . .    
417	G G G . . .    
418	A A A . . .    
419	C C C . . .    
420	C C C . . .    
421	C C C . . .    
422	A A A . . .    
423	A A A . . .    
424	C C C . . .    
425	C C C . . .    
426	C C C . . .    
427	T T T . . .    
428	C C C . . .    
429	A A A . . .    
430	A A A . . .    
431	A A A . . .    
432	G G G . . .    
433	G G G . . .    
434	G G G . . .    
435	T T T . . .    
436	C C C . . .    
437	C C C . . .    
438	C C C . . .    
439	C C C . . .    
440	A A A . . .    
441	A A A . . .    
442	G G G . . .    
443	A A A . . .    
444	C C C . . .    
445	C C C . . .    
446	A A A . . .    
447	C C C . . .    
448	C C C . . .    
449	C C C . . .    
450	A A A . . .    
451	G G G . . .    
452	T T T . . .    
453	C C C . . .    
454	C C C . . .    
455	C C C . . .    
456	G G G . . .    
457	C C C . . .    
458	A A A . . .    
459	G G G . . .    
460	A A A . . .    
461	T T T . . .    
462	C C C . . .    
463	T T T . . .    
464	C C C . . .    
465	G G G . . .    
466	G G G . . .    
467	T T T . . .    
468	G G G G . .    
469	A A A A . .    
470	A A A A . .    
471	G G G G G .    
472	A A A A A .    
473	T T T T T .    
474	G G G G G .    
475	G G G G G .    
476	T T T T T .    
477	C C C C C .    
478	T T T T T .    
479	A A A A A .    
480	C C C C C .    
481	T T T T T .    
482	T T T T T .    
483	A A A A A .    
484	T T T T T .    
485	C C C C C .    
486	G G G G G .    
487	G G G G G .    
488	G G G G G .    
489	C C C C C .    
490	T T T T T .    
491	A A A A A .    
492	G G G G G .    
493	A A A A A .    
494	C C C C C .    
495	T T T T T .    
496	G G G G G .    
497	G G G G G .    
498	A A A A A .    
499	C C C C C .    
500	C C C C C .    
501	G G G G G .    
502	A G G G G .    
503	. A A A A .    
504	. A A A A .    
505	. C C C C .    
506	. C C C C .    
507	. G G G G .    
508	. G G G G .    
509	. A A A A .    
510	. T T T T .    
511	. C C C C .    
512	. G G G G .    
513	. T T T T .    
514	. G G G G .    
515	. T T T T .    
516	. T T T T .    
517	. C C C C .    
518	. A A A A .    
519	. T T T T .    
520	. T T T T .    
521	. T T T T .    
522	. T T T T .    
523	. G G G G .    
524	. G G G G .    
525	. C C C C .    
526	. A A A A .    
527	. C C C C .    
528	. A A A A .    
529	. G G G G .    
530	. T T T T .    
531	. G G G G .    
532	. T T T T .    
533	. A A A A .    
534	. G G G G .    
535	. C C C C .    
536	. A A A A .    
537	. A A A A .    
538	. A A A A .    
539	. A A A A .    
540	. C C C C .    
541	. A A A A .    
542	. T T T T .    
543	. T T T T .    
544	. T T T T .    
545	. T T T T .    
546	. G G G G .    
547	. C C C C .    
548	. A A A A .    
549	. C C C C .    
550	. A A A A .    
551	. G G G G .    
552	. G G G G .    
553	. A A A A .    
554	. G G G G .    
555	. A A A A .    
556	. A A A A .    
557	. C C C C .    
558	. G G G G .    
559	. A A A A .    
560	. A A A A .    
561	. A A A A .    
562	. A A A A .    
563	. C C C C .    
564	. G G G G .    
565	. A A A A .    
566	. G G G G .    
567	. C C C C .    
568	. G G G G .    
569	. T T T T .    
570	. T T T T .    
571	. T T T T .    
572	. C C C C .    
573	. T T T T .    
574	. T T T T .    
575	. A A A A .    
576	. T T T T .    
577	. T T T T .    
578	. G G G G .    
579	. G G G G .    
580	. A A A A .    
581	. C C C C .    
582	. A A A A .    
583	. A A A A .    
584	. G G G G .    
585	. T T T T .    
586	. T T T T .    
587	. C C C C .    
588	. A A A A .    
589	. G G G G .    
590	. G G G G .    
591	. G G G G .    
592	. T T T T .    
593	. C C C C .    
594	. A A A A .    
595	. T T T T .    
596	. A A A A .    
597	. T T T T .    
598	. T T T T .    
599	. C C C C .    
600	. A A A A .    
601	. T T T T .    
602	. T T T T .    
603	. A A A A .    
604	. G G G G .    
605	. T T T T .    
606	. G G G G .    
607	. G G G G .    
608	. A A A A .    
609	. A A A A .    
610	. A A A A .    
611	. T T T T .    
612	. G G G G .    
613	. T T T T .    
614	. T T T T .    
615	. T T T T .    
616	. T T T T .    
617	. G G G G .    
618	. G G G G .    
619	. A A A A .    
620	. A A A A .    
621	. A A A A .    
622	. A A A A .    
623	. T T T T .    
624	. G G G G .    
625	. A A A A .    
626	. A A A A .    
627	. A A A A .    
628	. A A A A .    
629	. C C C C .    
630	. A A A A .    
631	. A A A A .    
632	. G G G G .    
633	. C C C C .    
634	. G G G G .    
635	. T T T T .    
636	. T T T T .    
637	. T T T T .    
638	. G G G G .    
639	. T T T T .    
640	. T T T T .    
641	. A A A A .    
642	. T T T T .    
643	. T T T T .    
644	. G G G G .    
645	. G G G G .    
646	. A A A A .    
647	. C C C C .    
648	. A A A A .    
649	. A A A A .    
650	. G G G G .    
651	. T T T T .    
652	. T T T T .    
653	. C C C C .    
654	. A A A A .    
655	. G G G G .    
656	. G G G G .    
657	. T T T T .    
658	. A A A A .    
659	. G G G G .    
660	. T T T T T    
661	. C C C C C    
662	. C C C C C    
663	. C C C C C    
664	. T T T T T    
665	. C C C C C    
666	. C C C C C    
667	. C C C C C    
668	. T T T T T    
669	. T T T T T    
670	. G G G G G    
671	. A A A A A    
672	. C C C C C    
673	. C C C C C    
674	. C C C C C    
675	. A A A A A    
676	. G G G G G    
677	. G G G G G    
678	. T T T T T    
679	. A A A A A    
680	. A A A A A    
681	. T T T T T    
682	. G G G G G    
683	. G A A A A    
684	. . A A A A    
685	. . T T T T    
686	. . A A A A    
687	. . C C C C    
688	. . T T T T    
689	. . G G G G    
690	. . T T T T    
691	. . T T T T    
692	. . G G G G    
693	. . A A A A    
694	. . T T T T    
695	. . A A A A    
696	. . C C C C    
697	. . C C C C    
698	. . T T T T    
699	. . A A A A    
700	. . A A A A    
701	. . A A A A    
702	. . A A A A    
703	. . A A A A    
704	. . G G G G    
705	. . A A A A    
706	. . T T T T    
707	. . A A A A    
708	. . T T T T    
709	. . A A A A    
710	. . C C C C    
711	. . A A A A    
712	. . C C C C    
713	. . A A A A    
714	. . C C C C    
715	. . A A A A    
716	. . C C C C    
717	. . A A A C    
718	. . C C C C    
719	. . A A A A    
720	. . G - G -  2	 indel  
721	. . A - A -  2	 indel  
722	. . A A A A    
723	. . T T T T    
724	. . C C C C    
725	. . C C C C    
726	. . A A A A    
727	. . T T T T    
728	. . C C C C    
729	. . T T T T    
730	. . C C C C    
731	. . A A A A    
732	. . . C C C    
733	. . . T T T    
734	. . . T T T    
735	. . . A A A    
736	. . . A A A    
737	. . . C C C    
738	. . . C C C    
739	. . . C C C    
740	. . . T T T    
741	. . . A A A    
742	. . . A A A    
743	. . . A A A    
744	. . . A A A    
745	. . . C C C    
746	. . . A A A    
747	. . . A A A    
748	. . . T T T    
749	. . . A A A    
750	. . . C C C    
751	. . . G G G    
752	. . . C C C    
753	. . . C C C    
754	. . . C C C    
755	. . . C C C    
756	. . . T T T    
757	. . . A A A    
758	. . . T T T    
759	. . . G G G    
760	. . . G G G    
761	. . . G G G    
762	. . . C C C    
763	. . . C C C    
764	. . . C C C    
765	. . . T T T    
766	. . . G G G    
767	. . . G G G    
768	. . . T T T    
769	. . . C C C    
770	. . . A A A    
771	. . . A A A    
772	. . . A A A    
773	. . . A A A    
774	. . . G G G    
775	. . . T T T    
776	. . . A A A    
777	. . . G G G    
778	. . . T T T    
779	. . . G G G    
780	. . . C C C    
781	. . . A A A    
782	. . . C C C    
783	. . . T T T    
784	. . . A A A    
785	. . . T T T    
786	. . . A A A    
787	. . . A A A    
788	. . . A A A    
789	. . . G G G    
790	. . . A A A    
791	. . . G G G    
792	. . . A A A    
793	. . . A A A    
794	. . . T T T    
795	. . . A A A    
796	. . . G G G    
797	. . . G G G    
798	. . . C C C    
799	. . . T T T    
800	. . . G G G    
801	. . . C C C    
802	. . . C C C    
803	. . . A A A    
804	. . . T T T    
805	. . . T T T    
806	. . . T T T    
807	. . . G G G    
808	. . . G G G    
809	. . . G G G    
810	. . . A A A    
811	. . . T T T    
812	. . . G G G    
813	. . . C C C    
814	. . . A A A    
815	. . . T T T    
816	. . . C C C    
817	. . . C C C    
818	. . . A A A    
819	. . . T T T    
820	. . . A A A    
821	. . . G G G    
822	. . . T T T    
823	. . . G G G    
824	. . . T T T    
825	. . . T T T    
826	. . . T T T    
827	. . . C C C    
828	. . . T T T    
829	. . . T T T    
830	. . . G G G    
831	. . . C C C    
832	. . . T T T    
833	. . . A A A    
834	. . . A A A    
835	. . . C C C    
836	. . . C C C    
837	. . . A A A    
838	. . . G G G    
839	. . . T T T    
840	. . . G G G    
841	. . . T T T    
842	. . . A A A    
843	. . . C C C    
844	. . . A A A    
845	. . . T T T    
846	. . . C C C    
847	. . . T T T    
848	. . . G G G    
849	. . . G G G    
850	. . . A A A    
851	. . . A A A    
852	. . . A A A    
853	. . . G G G    
854	. . . T T T    
855	. . . A A A    
856	. . . T T T    
857	. . . T T T    
858	. . . G G G    
859	. . . T T T    
860	. . . A A A    
861	. . . T T T    
862	. . . T T T    
863	. . . T T T    
864	. . . T T T    
865	. . . A A A    
866	. . . A A A    
867	. . . C C C    
868	. . . T T T    
869	. . . T T T    
870	. . . G G G    
871	. . . A A A    
872	. . . T T T    
873	. . . T T T    
874	. . . G G G    
875	. . . T T T    
876	. . . A A A    
877	. . . T T T    
878	. . . T T T    
879	. . . C C C    
880	. . . T T T    
881	. . . A A A    
882	. . . C C C    
883	. . . A A A    
884	. . . A A A    
885	. . . A A A    
886	. . . G G G    
887	. . . G G G    
888	. . . C C C    
889	. . . A A A    
890	. . . C C C    
891	. . . A A A    
892	. . . A A A    
893	. . . C C C    
894	. . . T T T    
895	. . . C C C    
896	. . . A A A    
897	. . . C C C    
898	. . . T T T    
899	. . . G G G    
900	. . . C C C    
901	. . . A A A    
902	. . . T T T    
903	. . . T T T    
904	. . . C C C    
905	. . . A A A    
906	. . . A A A    
907	. . . A A A    
908	. . . T T T    
909	. . . G G G    
910	. . . C C C    
911	. . . A A A    
912	. . . T T T    
913	. . . C C C    
914	. . . T T T    
915	. . . T T T    
916	. . . A A A    
917	. . . G G G    
918	. . . C C C    
919	. . . T T T    
920	. . . A A A    
921	. . . C C C    
922	. . . T T T    
923	. . . A A A    
924	. . . C C C    
925	. . . A A A    
926	. . . T T T    
927	. . . A A A    
928	. . . C C C    
929	. . . T T T    
930	. . . G G G    
931	. . . T T T    
932	. . . A A A    
933	. . . T T T    
934	. . . G G G    
935	. . . T T T    
936	. . . G G G    
937	. . . A A A    
938	. . . C C C    
939	. . . A A A    
940	. . . A A A    
941	. . . T T T    
942	. . . C C C    
943	. . . T T T    
944	. . . A A A    
945	. . . T T T    
946	. . . T T T    
947	. . . C C C    
948	. . . T T T    
949	. . . T T T    
950	. . . A A A    
951	. . . C C C    
952	. . . C C C    
953	. . . A A A    
954	. . . C C C    
955	. . . A A A    
956	. . . A A A    
957	. . . G G G    
958	. . . A A A    
959	. . . C C C    
960	. . . C C C    
961	. . . T T T    
962	. . . C C C    
963	. . . A A A    
964	. . . G G G    
965	. . . G G G    
966	. . . A A A    
967	. . . C C C    
968	. . . T T T    
969	. . . T T T    
970	. . . G G G    
971	. . . T T T    
972	. . . T T T    
973	. . . T T T    
974	. . . T T T    
975	. . . T T T    
976	. . . G G G    
977	. . . G G G    
978	. . . A A A    
979	. . . C C C    
980	. . . A A A    
981	. . . C C C    
982	. . . A A A    
983	. . . C C C    
984	. . . T T T    
985	. . . G G G    
986	. . . T T T    
987	. . . T T T    
988	. . . C C C    
989	. . . G G G    
990	. . . T T T    
991	. . . A A A    
992	. . . A A A    
993	. . . T T T    
994	. . . A A A    
995	. . . T T T    
996	. . . G G G    
997	. . . T T T    
998	. . . T T T    
999	. . . A A A    
1000	. . . G G G    
1001	. . . T T T    
1002	. . . T T T    
1003	. . . T T T    
1004	. . . T T T    
1005	. . . A A A    
1006	. . . C C C    
1007	. . . A A A    
1008	. . . C C C    
1009	. . . A A A    
1010	. . . T T T    
1011	. . . T T T    
1012	. . . T T T    
1013	. . . T T T    
1014	. . . A A A    
1015	. . . T T T    
1016	. . . A A A    
1017	. . . C C C    
1018	. . . T T T    
1019	. . . A A A    
1020	. . . T T T    
1021	. . . T T T    
1022	. . . T T T    
1023	. . . A A A    
1024	. . . T T T    
1025	. . . A A A    
1026	. . . T T T    
1027	. . . T T T    
1028	. . . T T T    
1029	. . . C C C    
1030	. . . C C C    
1031	. . . T T T    
1032	. . . A A A    
1033	. . . T T T    
1034	. . . A A A    
1035	. . . C C C    
1036	. . . C C C    
1037	. . . T T T    
1038	. . . G G G    
1039	. . . T T T    
1040	. . . T T T    
1041	. . . T T T    
1042	. . . G G G    
1043	. . . T T T    
1044	. . . G G G    
1045	. . . G G G    
1046	. . . T T T    
1047	. . . A A A    
1048	. . . C C C    
1049	. . . A A A    
1050	. . . G G G    
1051	. . . T T T    
1052	. . . A A A    
1053	. . . T T T    
1054	. . . T T T    
1055	. . . G G G    
1056	. . . G G G    
1057	. . . G G G    
1058	. . . A A A    
1059	. . . T T T    
1060	. . . G G G    
1061	. . . G G G    
1062	. . . C C C    
1063	. . . A A A    
1064	. . . A A A    
1065	. . . T T T    
1066	. . . G G G    
1067	. . . C C C    
1068	. . . A A A    
1069	. . . A A A    
1070	. . . C C C    
1071	. . . C C C    
1072	. . . A A A    
1073	. . . A A A    
1074	. . . A A A    
1075	. . . G G G    
1076	. . . C C C    
1077	. . . C C C    
1078	. . . C C C    
1079	. . . A A A    
1080	. . . G G G    
1081	. . . A A A    
1082	. . . A A A    
1083	. . . T T T    
1084	. . . A A A    
1085	. . . T T T    
1086	. . . C C C    
1087	. . . C C C    
1088	. . . A A A    
1089	. . . A A A    
1090	. . . G G G    
1091	. . . T T T    
1092	. . . C C C    
1093	. . . T T T    
1094	. . . A A A    
1095	. . . T T T    
1096	. . . T T T    
1097	. . . T T T    
1098	. . . T T T    
1099	. . . T T T    
1100	. . . A A A    
1101	. . . A A A    
1102	. . . G G G    
1103	. . . G G G    
1104	. . . T T T    
1105	. . . C C C    
1106	. . . T T T    
1107	. . . G G G    
1108	. . . T T T    
1109	. . . T T T    
1110	. . . T T T    
1111	. . . T T T    
1112	. . . T T T    
1113	. . . A A A    
1114	. . . G G G    
1115	. . . T T T    
1116	. . . G G G    
1117	. . . T T T    
1118	. . . T T T    
1119	. . . C C C    
1120	. . . T T T    
1121	. . . T T T    
1122	. . . C C C    
1123	. . . T T T    
1124	. . . T T T    
1125	. . . T T T    
1126	. . . C C C    
1127	. . . C C C    
1128	. . . T T T    
1129	. . . A A A    
1130	. . . C C C    
1131	. . . T T T    
1132	. . . G G G    
1133	. . . T T T    
1134	. . . T T T    
1135	. . . T T T    
1136	. . . T T T    
1137	. . . T T T    
1138	. . . G G G    
1139	. . . T T T    
1140	. . . T T T    
1141	. . . C C C    
1142	. . . T T T    
1143	. . . A A A    
1144	. . . A A A    
1145	. . . A A A    
1146	. . . T T T    
1147	. . . T T T    
1148	. . . A A A    
1149	. . . T T T    
1150	. . . T T T    
1151	. . . T T T    
1152	. . . A A A    
1153	. . . C C C    
1154	. . . A A A    
1155	. . . A A A    
1156	. . . G G G    
1157	. . . T T T    
1158	. . . G G G    
1159	. . . T T T    
1160	. . . T T T    
1161	. . . T T T    
1162	. . . T T T    
1163	. . . T T T    
1164	. . . T T T    
1165	. . . A A A    
1166	. . . A A A    
1167	. . . T T T    
1168	. . . A A A    
1169	. . . A A A    
1170	. . . A A A    
1171	. . . T T T    
1172	. . . G G G    
1173	. . . T T T    
1174	. . . T T T    
1175	. . . T T T    
1176	. . . T T T    
1177	. . . T T T    
1178	. . . A A A    
1179	. . . T T T
```
